# Supplementary material for: Habitat Characteristics Coincidence of Dead and Living Long-Tailed Gorals (Naemorhedus caudatus) According to Extreme Snowfall
Source: Animals (Basel). 2021 Apr 2;11(4):997. doi: 10.3390/ani11040997 (PMC8067025; doi:10.3390/ani11040997)
Supplement: Supplementary file 1 [file animals-11-00997-s001.pdf]

Habitat Characteristics Coincidence of Dead and Living Long-tailed Gorals (*Naemorhedus Caudatus*) according to Extreme Snowfall

Hee-Bok Park[1], Sungwon Hong[2,3*]

[1] Restoration Research Team (mammals), Research Center for Endangered Species, National Institute of Ecology, Yeongyang 36531, Republic of Korea

[2] Department of Animal Science and Biotechnology, Kyungpook National University, Sangju 37224, Republic of Korea

[3] Department of Horse, Companion, and Wild Animal Science, Kyungpook National University, Sangju 37224, Republic of Korea

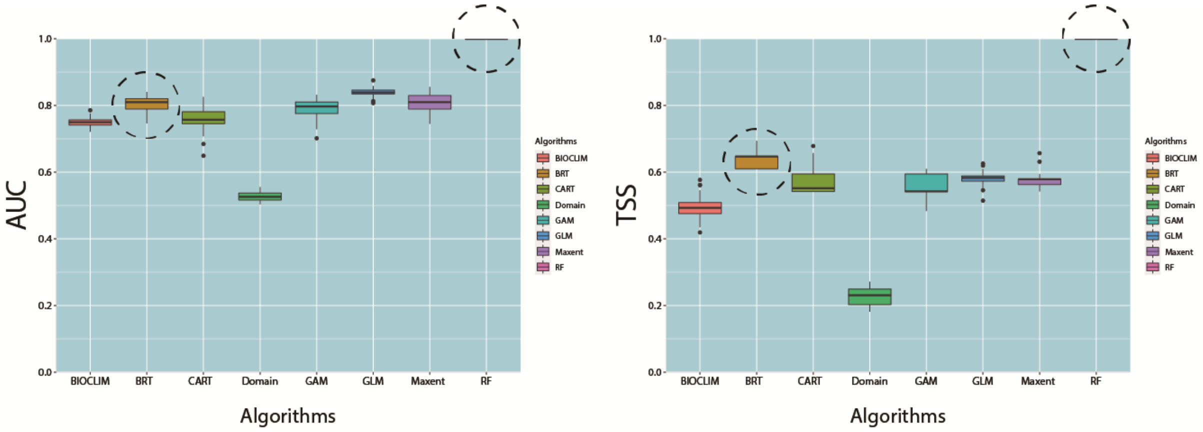

Appendix A.1. Box plots of the average and standard error of area under the ROC curve (AUC) and True Skill Statistic (TSS) values of 8 model algorithms (BIOCLIM, Domain, generalised linear models (GLMs), generalised additive models (GAMs), classification and regression tree (CART), random forests (RFs), boosted regression trees (BRTs), and Maxent) to predict goral habitats. The dotted circles indicate the selected models of the 8 models using the ensemble model.

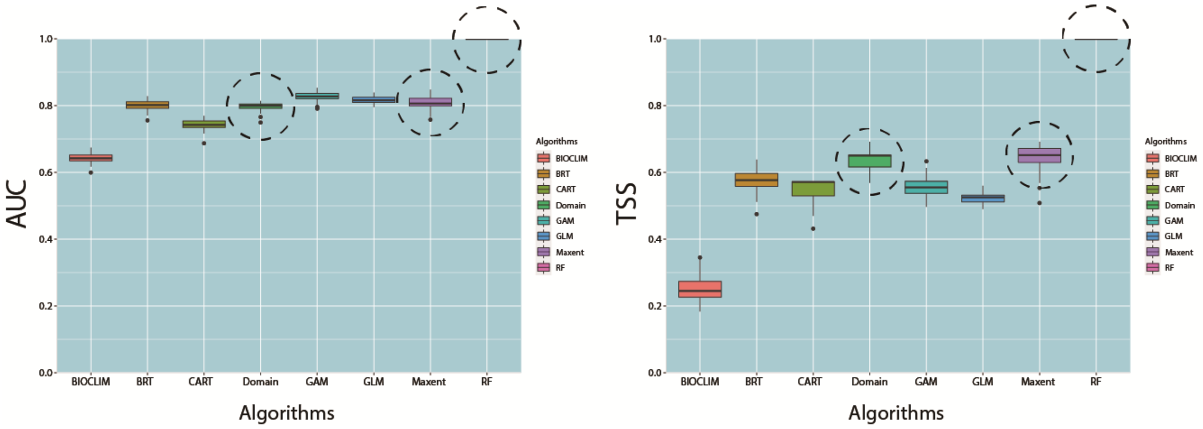

Appendix A.2. Box plots of the average and standard error of area under the ROC curve (AUC) and True Skill Statistic (TSS) values of 8 model algorithms (BIOCLIM, Domain, Generalised linear models (GLMs), Generalised additive models (GAMs), Classification and regression tree (CART), Random Forests (RFs), Boosted regression trees (BRTs), and Maxent) to predict areas with high mortality rate due to heavy snowfall. Please see my above comment.
